# Gut Microbiome Composition and Response to Immune Checkpoint Inhibitors in Melanoma: A Systematic Review

**DOI:** 10.7759/cureus.113248

**Published:** 2026-07-23

**Authors:** Keemaya G Joglekar

**Affiliations:** 1 Research, Independent Researcher, Mumbai, IND

**Keywords:** gut microbiome, immune checkpoint inhibitors, immunotherapy, melanoma, microbiome diversity, pd-1 blockade, systematic review, tumor immunity

## Abstract

Immune checkpoint inhibitors have significantly improved clinical outcomes in patients with advanced melanoma; however, treatment response remains highly variable, and reliable predictive biomarkers are lacking. Emerging evidence suggests that the gut microbiome influences host immune regulation and may contribute to this variability. This systematic review evaluated the association between gut microbiome composition and response to immune checkpoint inhibitors in melanoma, with a focus on potential biological mechanisms, predictive biomarkers, and therapeutic implications. A systematic search of the PubMed database was conducted to identify studies published between 2015 and 2025. Eligible studies included original observational investigations and secondary analyses of melanoma microbiome datasets that examined associations between gut microbiome characteristics and response to immune checkpoint inhibitors. Interventional fecal microbiota transplantation studies, dietary or probiotic intervention trials, exposure-only studies (including antibiotic, proton pump inhibitor, or *Helicobacter pylori* exposure without direct microbiome sequencing), non-English-language publications, and preprints were excluded. The search identified 381 records. After removal of one duplicate, 380 records were screened, and 25 studies met the inclusion criteria. Across the included studies, gut microbiome composition, microbial diversity, and metabolic function were associated with immunotherapy outcomes. Increased abundance of short-chain fatty acid-producing taxa, including *Faecalibacterium prausnitzii* and *Akkermansia muciniphila*, was frequently associated with improved treatment response, whereas dysbiosis and enrichment of pathogenic bacterial and fungal taxa were linked to reduced therapeutic efficacy and poorer survival outcomes. Microbiome-derived metabolites appear to modulate antitumor immunity through effects on dendritic cell function, antigen presentation, and T-cell activation, while longitudinal alterations in microbiome composition may serve as early indicators of treatment response. Overall, findings were heterogeneous because of differences in patient populations, sequencing methodologies, outcome definitions, and analytical approaches. Risk-of-bias assessment using the Risk Of Bias In Non-randomized Studies of Interventions (ROBINS-I) tool rated 17 of the 25 included studies as having a moderate risk of bias and eight as having a serious risk of bias, primarily because of residual confounding and small single-center study designs. The gut microbiome represents a promising predictive biomarker and potential therapeutic target for melanoma immunotherapy; however, substantial methodological heterogeneity limits current clinical applicability. Large, standardized, prospective, multicenter, multi-omic studies are needed before microbiome-guided treatment strategies can be incorporated into routine clinical practice.

## Introduction and background

Melanoma is among the most aggressive forms of skin cancer. Although immune checkpoint inhibitors have significantly improved clinical outcomes, only a subset of patients achieves durable responses, and understanding the determinants of therapeutic response remains a major challenge in oncology. Emerging evidence suggests that the gut microbiome plays a role in modulating both melanoma progression and response to immunotherapy.

Immune checkpoint inhibitors are monoclonal antibodies that restore antitumor T-cell activity by blocking inhibitory signaling, principally through programmed cell death protein 1 (PD-1) and its ligand, i.e., programmed death-ligand 1 (PD-L1), and cytotoxic T-lymphocyte-associated protein 4 (CTLA-4). The gut microbiome, the community of microorganisms inhabiting the intestinal tract, helps shape systemic immune responses, partly through microbial metabolites such as short-chain fatty acids (SCFAs) that support immune regulation. A disruption of this microbial balance is termed dysbiosis. As durable responses occur in only a subset of patients and reliable predictive biomarkers are lacking, identifying reproducible microbiome-based biomarkers could help stratify patients likely to benefit and guide microbiome-directed treatment strategies, which is the central gap this review addresses.

Greater gut microbial diversity and enrichment of specific taxa, including *Ruminococcaceae*, *Bifidobacterium*, and *Akkermansia*, have been consistently associated with improved response to immune checkpoint inhibitors [1-3]. In contrast, enrichment of taxa, including *Peptostreptococcaceae*, *Malassezia restricta*, and *Candida albicans*, has been linked to poor therapeutic outcomes [4,5]. Members of the *Lachnospiraceae* family have been associated with both improved treatment response and reduced immune-related adverse events, potentially mediated by flagellin-related pathways that enhance antitumor immunity [6-8].

Metagenomic analyses demonstrate differences in microbial composition between responders and non-responders; for example, the *Bacteroidota-to-Firmicutes* ratio has been reported to be higher in responders [9]. Dietary factors also appear to influence microbiome composition and treatment outcomes, with distinct dietary patterns observed among patients with delayed response [10], and microbial signatures involving *Bacteroides* species have been implicated in predicting immune-related adverse events [11].

Collectively, these findings position the gut microbiome as a modulator of immunotherapy outcomes in melanoma. This review synthesizes current evidence on the relationship between gut microbiota composition and response to immune checkpoint inhibitors in melanoma, and evaluates the methodological quality of the underlying evidence base.

## Review

Methodology

A systematic literature search of the PubMed database was conducted in July 2025 using combinations of Medical Subject Headings (MeSH) and free-text terms related to melanoma, the gut microbiome, and immune checkpoint inhibitors: (“Melanoma”[MeSH] OR melanoma[All Fields]) AND (“Microbiota”[MeSH] OR microbiome[All Fields] OR gut microbiome[All Fields]) AND (“Immunotherapy”[MeSH] OR “Immune Checkpoint Inhibitors”[MeSH] OR “PD-1”[All Fields] OR “PD-L1”[All Fields] OR “CTLA-4”[All Fields]). Only the PubMed database was included in the search strategy.

Studies were included if they investigated melanoma as a primary focus or reported melanoma-specific data separately within a multi-cancer population, and examined the relationship between gut microbiome composition or function and response to immune checkpoint inhibitors (anti-PD-1, anti-PD-L1, or anti-CTLA-4) as a primary variable of interest, with a reported clinical outcome. Original observational studies and secondary analyses of eligible melanoma microbiome datasets were included, provided they investigated associations between gut microbiome characteristics and immune checkpoint inhibitor response. All included studies were original, English-language, peer-reviewed research published between 2015 and 2025. Studies were excluded if they were reviews, meta-analyses, conference abstracts, protocol papers, editorials, or opinion articles without original data; involved non-human models; lacked melanoma-specific or sufficient clinical outcome data; or were preprints that had not undergone peer review. Interventional fecal microbiota transplantation trials, dietary or probiotic intervention trials, and exposure-only studies (antibiotic, proton pump inhibitor, or *Helicobacter pylori* exposure without gut microbiome sequencing as the primary analysis) were excluded to restrict this review to observational studies directly measuring gut microbiome composition.

The search yielded 381 records. After removal of one duplicate, 380 records were screened by title and abstract. Following initial screening, 68 full-text articles were assessed for eligibility. Of these, 43 were excluded for reasons including absence of melanoma-specific data, review or commentary articles, animal studies, non-English publications, lack of gut microbiome as a primary variable, inappropriate study design, in vitro studies, or wrong cancer type. Ultimately, 25 studies met all inclusion criteria and were included in the qualitative synthesis.

**Figure 1:**
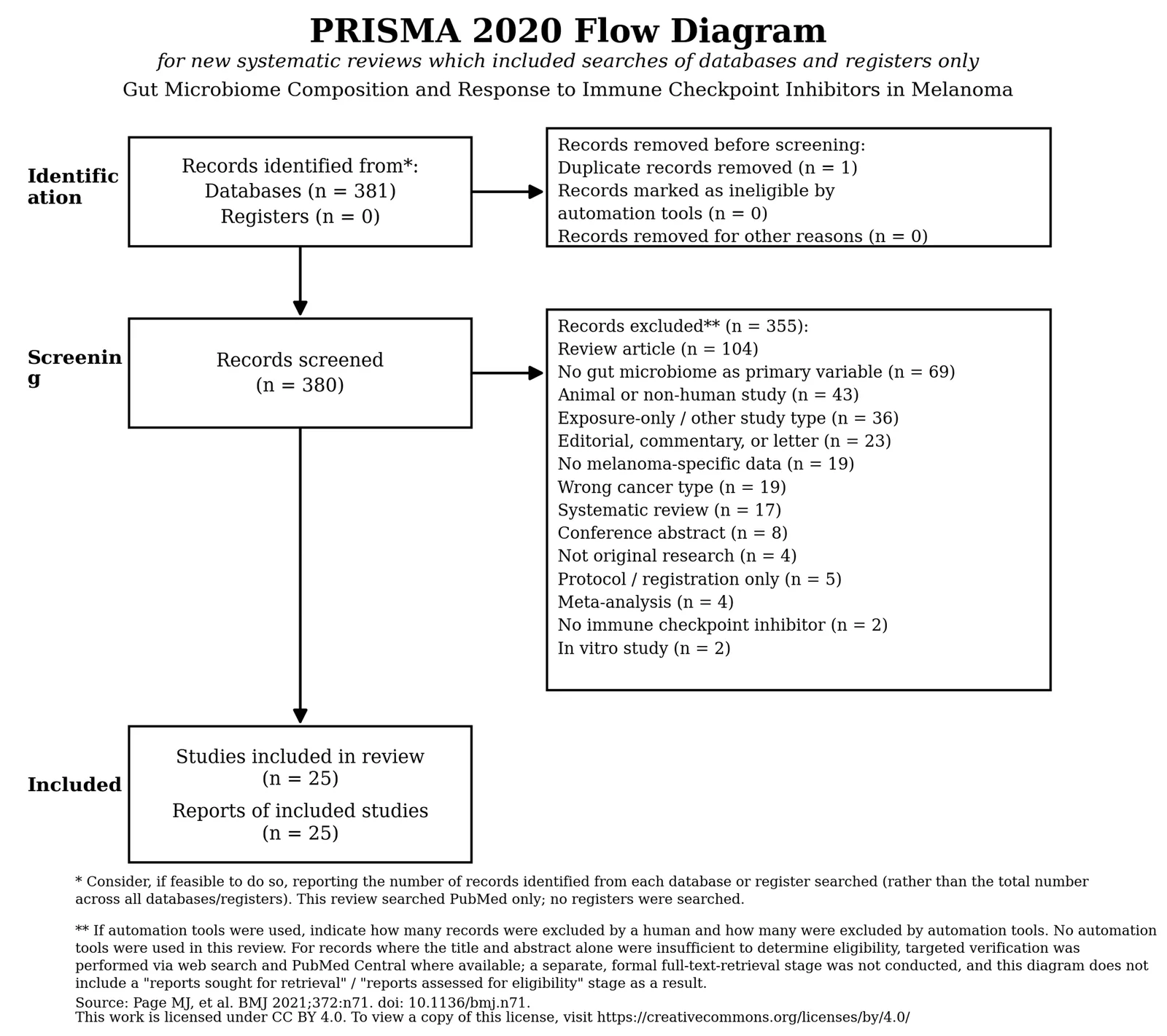
PRISMA 2020 flow diagram of study identification, screening, and inclusion.

This review followed the Preferred Reporting Items for Systematic Reviews and Meta-Analyses (PRISMA) 2020 guidelines (Figure 1) [12]. Risk of bias in the included observational studies was assessed using the Risk Of Bias In Non-randomized Studies of Interventions (ROBINS-I) tool, described further below [13]. This systematic review was not prospectively registered in a protocol registry such as the International Prospective Register of Systematic Reviews (PROSPERO). Study selection, data extraction, and risk-of-bias assessment were conducted by a single reviewer using predefined eligibility and extraction criteria. A narrative (qualitative) synthesis was performed rather than a meta-analysis because substantial heterogeneity across the included studies in sequencing platforms, taxonomic classification, outcome definitions, and analytical pipelines precluded valid quantitative pooling; consequently, no pooled effect estimates, P-values, or confidence intervals were generated.

Inclusion Criteria

Studies were included if they investigated melanoma as a primary focus or provided melanoma-specific data in human populations. Eligible studies examined the relationship between gut microbiome composition or function and response to immune checkpoint inhibitors (anti-PD-1, anti-PD-L1, or anti-CTLA-4). Only studies in which the gut microbiome was a primary variable of interest were included. Primary research articles with full-text availability in English, published between 2015 and 2025, were considered.

Exclusion Criteria

Studies were excluded if they did not address melanoma in conjunction with gut microbiome composition and immune checkpoint inhibitor therapy, lacked sufficient clinical outcome data, or were reviews, meta-analyses, conference abstracts, protocol studies, or opinion articles without original data. Non-human studies were also excluded.

Data Extraction and Quality Assessment

Data were extracted using a standardized form capturing study design, sample size, type of immunotherapy, microbiome analysis methods, key findings, and study limitations. Study selection and data extraction were performed systematically using predefined criteria.

A formal risk of bias assessment was conducted for all included studies using the ROBINS-I tool [13]. Although ROBINS-I was originally developed for non-randomized studies of interventions, it was considered appropriate because the included observational studies evaluated naturally occurring microbiome characteristics as exposures and required structured assessment of confounding, participant selection, and outcome measurement. This tool was selected due to the observational and non-randomized nature of the included studies. Each study was evaluated across the domains of bias due to confounding, selection of participants, classification of exposures, deviations from intended exposures, missing data, measurement of outcomes, and selection of the reported result. Based on these domains, each study was assigned an overall risk of bias rating categorized as low, moderate, serious, or critical.

Results

Twenty-five original studies published between 2015 and 2025 met the inclusion criteria (Table 1). Sample sizes ranged from 23 to 680 participants. Most studies evaluated anti-PD-1 therapy, whereas a smaller number investigated ipilimumab monotherapy or combination anti-PD-1/anti-CTLA-4 regimens.

**Table 1:** Characteristics of included studies evaluating the gut microbiome and response to immune checkpoint inhibitors in melanoma. ICI: immune checkpoint inhibitor; PD-1: programmed cell death protein 1; CTLA-4: cytotoxic T-lymphocyte-associated protein 4; irAE: immune-related adverse event; CICB: combination immune checkpoint blockade; NSCLC: non-small cell lung cancer; SCFA: short-chain fatty acid.

Study	Sample size	Immunotherapy	Key microbiome findings	Main outcome
Gopalakrishnan et al. (2018) [1]	112 (43 fecal)	Anti-PD-1	Higher alpha diversity and *Ruminococcaceae* abundance in responders; enrichment of anabolic pathways	Gut and oral microbiome diversity associated with anti-PD-1 response
Matson et al. (2018) [14]	42	Anti-PD-1	Higher *Bifidobacterium longum*, *Collinsella aerofaciens*, and *Enterococcus faecium* abundance in responders	Baseline commensal microbiome composition associated with anti-PD-1 efficacy
Lee et al. (2022) [2]	165 (+147 external)	ICI	Cohort-dependent gut microbiome-response association; panel including *Bifidobacterium pseudocatenulatum*, *Roseburia *spp., and *Akkermansia muciniphila*	Cross-cohort gut microbiome associations with ICI response
McCulloch et al. (2022) [8]	New cohort plus four pooled datasets	Anti-PD-1	*Actinobacteria*, *Lachnospiraceae*, and *Ruminococcaceae* favorable; *Streptococcaceae* unfavorable, with distinct irAEs	Intestinal microbiota signatures predict response and irAEs across cohorts
Andrews et al. (2021) [7]	77	CTLA-4 + PD-1 (CICB)	Higher *Bacteroides intestinalis* abundance associated with toxicity (grade ≥3 irAEs in 49% of patients)	Gut microbiota signatures associated with CICB toxicity
Zhou et al. (2025) [3]	>200	ICI	Distinct bacterial and fungal biomarkers with interaction networks associated with response	Multi-omics microbiome signatures predictive of response
Szóstak et al. (2025) [4]	61	Anti-PD-1	Reduced fungal diversity; *Candida* and *Malassezia* enrichment in poorer outcomes	Gut mycobiome composition correlated with response
Macandog et al. (2024) [6]	23	Anti-PD-1	Stable microbial functional profiles and flagellin-related peptides in complete responders	Longitudinal microbiome stability associated with response
Björk et al. (2024) [15]	175	ICB	Species-level genome bins distinguish progression-free survival ≥12 versus <12 months	Longitudinal gut microbiome changes associated with progression-free survival
Pietrzak et al. (2022) [9]	74	Anti-PD-1	Higher *Bacteroidota-to-Firmicutes* ratio in responders	Composition associated with treatment response
Simpson et al. (2022) [10]	218 (103 from Australia and Netherlands, 115 from the United States)	Neoadjuvant ICI	*Ruminococcaceae*-dominated microbiomes associated with higher response; low fiber and omega-3 intake and elevated C-reactive protein associated with poor response	Diet-driven microbial ecology underpins ICI outcomes
Wind et al. (2020) [5]	25	ICI	No alpha-diversity difference between groups; 68 taxa differentially abundant after adjustment for age, body mass index, and antibiotic use	Confounder-adjusted microbial species and pathways associated with response
Usyk et al. (2021) [11]	27	ICB (ipilimumab + nivolumab)	Two gut microbiome clusters identified (*Bacteroides dorei*-high versus *Bacteroides vulgatus*-high); *B. dorei*-high cluster had approximately 7-fold higher irAE risk	Gut microbiome predicts immune-related adverse events
Szóstak et al. (2024) [16]	220	Anti-PD-1	Fungal dysbiosis characterized by increased *Candida* and decreased *Saccharomyces* abundance	Mycobiome alterations associated with progression and response
Zakharevich et al. (2024) [17]	680	ICI	Cobalamin and short-chain fatty acid metabolic pathways associated with response	Functional metabolic signatures predictive of efficacy
Olekhnovich et al. (2023) [18]	680 (reanalysis of seven studies)	Mixed ICI	*Faecalibacterium prausnitzii*, *Bifidobacterium adolescentis*, and *Eubacterium rectale* identified as consistent cross-study biomarkers	Consistent stool metagenomic biomarkers of response
Tsakmaklis et al. (2023) [19]	29	PD-1 ± CTLA-4	Combined TIGIT+ natural killer cell profiles and gut microbiota features associated with response	Integrated immune-microbiome model predictive of outcomes
Bredon et al. (2024) [20]	Two human cohorts (NSCLC and melanoma) plus a mouse model	Anti-PD-1	High baseline *F. prausnitzii* abundance associated with better response in humans; strain EXL01 restored response in antibiotic-perturbed mice	Baseline *F. prausnitzii* abundance associated with response; causal support from a mouse model
Chaput et al. (2017) [21]	26	Ipilimumab (CTLA-4)	*Faecalibacterium*/*Firmicutes*-dominant cluster associated with longer progression-free and overall survival but higher colitis risk; *Bacteroides*-dominant cluster associated with colitis resistance	Baseline gut microbiota predicts response and colitis
Sardar et al. (2025) [22]	112	Anti-PD-1	Hexa-acylated lipopolysaccharide-producing bacteria enriched in responders	Immunostimulatory lipopolysaccharide-producing bacteria predict response
Drymel et al. (2025) [23]	64 (115 samples)	Anti-PD-1	Intestinal barrier markers (zonulin, calprotectin, secretory immunoglobulin A) characterized alongside stool microbiota	Barrier state and stool microbiota associated with outcomes
Peters et al. (2019) [24]	27 (17 in the metatranscriptome subset)	Anti-PD-1	Higher microbial richness associated with longer progression-free survival; L-rhamnose degradation, guanosine, and B-vitamin biosynthesis pathways linked to progression-free survival	Gut metagenome and metatranscriptome associated with response
Oh and Zhang (2023) [25]	Uses published melanoma ICI microbiome datasets	Anti-PD-1 (secondary analysis)	Deep-learning autoencoder (DeepGeni) identifies taxa informative for ICI response, linked to progression-free survival and responder status	Machine-learning-based prediction of ICI response from gut microbiome profiles
Ou et al. (2025) [26]	80	Immunotherapy (mixed regimens)	Quantitative polymerase chain reaction-based panel of 41 markers; low *Akkermansia muciniphila* and high *Bacteroides vulgatus* abundance associated with good response	Quantitative polymerase chain reaction gut marker panel predicts immunotherapy response
Frankel et al. (2017) [27]	39	Ipilimumab, nivolumab, ipilimumab + nivolumab, or pembrolizumab	*Bacteroides caccae* enriched in responders across regimens; *Faecalibacterium prausnitzii*, *Bacteroides thetaiotaomicron*, and *Holdemania filiformis* in ipilimumab + nivolumab responders; high anacardic acid	Pretreatment gut microbiome and metabolites associated with immune checkpoint therapy response (first prospective human study)

Associations between microbial diversity and treatment response were heterogeneous. Some studies reported lower Shannon diversity and disrupted community structure in non-responders [4,16], while another found lower microbial richness in responders despite a higher *Bacteroidota-to-Firmicutes* ratio [9], and a confounder-adjusted analysis found no significant alpha-diversity difference despite 68 differentially abundant taxa [5], indicating that diversity alone is not a reliable standalone biomarker of response. Large-scale cohort data supported a broader role for overall composition, with depletion of beneficial taxa and enrichment of pathobionts correlating with worse outcomes [17].

Responders were frequently enriched in taxa linked to metabolic and immune-modulatory function, including *Ruminococcaceae* and *Lachnospiraceae* [6-8], while non-responders showed enrichment of *Candida albicans* and *Malassezia restricta* [16]. Associations were not always consistent at the individual-taxon level: *Faecalibacterium prausnitzii*, typically considered beneficial, was associated with non-response in one cohort [9], while a large multi-cohort reanalysis identified it as a consistent positive biomarker [18] and a related study used an *F. prausnitzii*-derived strain to enhance response [20], highlighting the strain-level, context-dependent nature of microbiome-host interactions (Table 2).

**Table 2:** Reported gut microbiome patterns associated with immune checkpoint inhibitor response in melanoma. SCFA: short-chain fatty acid; CRP: C-reactive protein.

Microbial feature or taxon	Association	Mechanistic insight	References
SCFA-producing *Firmicutes* (e.g., *F. prausnitzii*)	Positive	Promotes epithelial barrier integrity, dendritic cell maturation, and CD8+ T-cell activation	[1,17,20]
Akkermansia muciniphila	Positive	Supports antitumor immunity; loss during therapy linked to emerging resistance	[1,2,15]
Lachnospiraceae	Positive	Associated with improved response and fewer immune-related adverse events	[6-8]
*Bacteroidales* (e.g., *B. dorei*)	Negative	Linked to immune-related adverse events and dysbiosis	[11]
*Candida albicans*, *Malassezia* spp.	Negative	Associated with fungal dysbiosis and poorer treatment response	[4,16]
High microbial diversity	Positive (heterogeneous)	Associated with improved response in some cohorts; not consistently reproduced	[4,9]
Fiber-rich or low omega-3 diet	Positive (fiber)/negative (low omega-3)	Fiber promotes beneficial SCFA producers; low fiber, low omega-3, and elevated CRP linked to poor response	[10]
Antibiotic use	Negative	Reduces microbial diversity and impairs immunotherapy efficacy	[1]

Functional and metabolic pathway analyses identified additional associations with treatment response: SCFA production and cobalamin-related pathways were associated with improved outcomes [17,18]. Administration of a defined *F. prausnitzii* strain restored anti-tumor response to checkpoint blockade in antibiotic-perturbed mice, complementing the human observational finding that higher baseline *F. prausnitzii* abundance is independently associated with better clinical response [20]. Diet and antibiotic exposure also influenced microbiome composition and outcomes [9,19], though these findings were variable and often limited by small sample sizes. Across studies, responders to anti-PD-1 therapy consistently showed more favorable microbiome and metabolome profiles, while dysbiosis and pathogenic enrichment were associated with reduced survival [4,9,17].

Risk of bias assessment

A formal risk-of-bias assessment was conducted using the ROBINS-I tool (Table 3). Although ROBINS-I was originally developed for non-randomized studies of interventions, it was considered appropriate because the included observational studies evaluated naturally occurring gut microbiome characteristics as exposures and required structured assessment of confounding, participant selection, exposure classification, and outcome measurement. Each study was evaluated across the domains of bias due to confounding, selection of participants, classification of exposures, deviations from intended exposures, missing data, outcome measurement, and selection of the reported result. An overall risk-of-bias rating of low, moderate, serious, or critical was assigned in accordance with ROBINS-I guidance.

**Table 3:** Risk of bias assessment of included studies using the ROBINS-I tool. Overall rating follows the standard Risk Of Bias In Non-randomized Studies of Interventions (ROBINS-I) convention: the least favorable (highest-risk) domain determines the overall rating.

Study	Confounding	Selection	Classification of exposure	Missing data	Outcome measurement	Reporting bias	Overall
Gopalakrishnan et al. (2018) [1]	Moderate	Moderate	Low	Low	Low	Low	Moderate
Matson et al. (2018) [14]	Moderate	Moderate	Moderate	Low	Low	Low	Moderate
Lee et al. (2022) [2]	Moderate	Low	Low	Low	Low	Low	Moderate
McCulloch et al. (2022) [8]	Moderate	Moderate	Moderate	Moderate	Low	Low	Moderate
Andrews et al. (2021) [7]	Moderate	Moderate	Low-moderate	Moderate	Low	Low	Moderate
Zhou et al. (2025) [3]	Moderate	Moderate	Low	Moderate	Moderate	Moderate	Moderate
Szóstak et al. (2025) [4]	Moderate	Moderate	Moderate	Low	Moderate	Low	Moderate
Macandog et al. (2024) [6]	Moderate	Serious	Low	Moderate	Low	Low	Serious
Björk et al. (2024) [15]	Moderate	Low	Low	Moderate	Low	Low	Moderate
Pietrzak et al. (2022) [9]	Moderate	Moderate	Moderate	Low	Low	Low	Moderate
Simpson et al. (2022) [10]	Low-moderate	Low	Low	Low	Low	Low	Moderate
Wind et al. (2020) [5]	Moderate	Serious	Low	Low	Moderate	Low	Serious
Usyk et al. (2021) [11]	Moderate	Serious	Low	Low	Low	Low	Serious
Szóstak et al. (2024) [16]	Moderate	Moderate	Moderate	Low	Moderate	Low	Moderate
Zakharevich et al. (2024) [17]	Moderate	Low	Low	Low	Moderate	Low	Moderate
Olekhnovich et al. (2023) [18]	Moderate	Moderate	Moderate	Moderate	Moderate	Moderate	Moderate
Tsakmaklis et al. (2023) [19]	Moderate	Serious	Moderate	Moderate	Low	Low	Serious
Bredon et al. (2024) [20]	Moderate	Moderate	Low	Moderate	Low	Low	Moderate
Chaput et al. (2017) [21]	Moderate	Serious	Moderate	Moderate	Low	Low	Serious
Sardar et al. (2025) [22]	Moderate	Moderate	Low	Low	Low	Low	Moderate
Drymel et al. (2025) [23]	Moderate	Moderate	Moderate	Low	Low	Low	Moderate
Peters et al. (2019) [24]	Moderate	Serious	Low	Moderate	Low	Low	Serious
Oh and Zhang (2023) [25]	Serious	Moderate	Moderate	Moderate	Low	Moderate	Serious
Ou et al. (2025) [26]	Moderate	Moderate	Moderate	Low	Low	Low	Moderate
Frankel et al. (2017) [27]	Moderate	Serious	Low	Moderate	Low	Low	Serious

Overall, 17 of 25 studies (68.0%) were rated moderate risk of bias, and eight of 25 (32.0%) were rated serious risk of bias. Seven of the eight serious ratings were driven by small, single-center sample sizes (the selection domain), and one (a secondary machine-learning reanalysis) by compounded confounding inherited from the underlying primary datasets. No study was rated low risk, since all are observational and some residual confounding is unavoidable in this design, and no study was rated critical risk, since every included study incorporated at least some adjustment, validation, or methodologically rigorous analysis. These ratings were based on assessment of the full text of each included study.

Discussion

Current evidence suggests that gut microbiome composition influences both the efficacy and toxicity of immune checkpoint blockade; however, no single microbial signature consistently predicts treatment response across all patient cohorts. Enrichment of SCFA-producing taxa, including *F. prausnitzii*, *Akkermansia muciniphila*, and *Bifidobacterium*, is associated with improved outcomes in several cohorts [1,2,20], though associations are not uniform, reflecting the context-dependent nature of microbiome-host interactions.

Mechanistic Insights

Mechanistically, SCFAs such as butyrate and propionate enhance dendritic cell maturation, promote antigen presentation, and stimulate cytotoxic CD8+ T-cell responses, partly through increased interferon-gamma production [28]; microbiota-derived metabolites more broadly modulate CD8+ T-cell responses and antitumor immunity [29]. Preclinical evidence in colorectal cancer mouse models further suggests that *Clostridiales* species can enhance antitumor immune responses and improve immunotherapy efficacy [30]; although this mechanism has not yet been directly demonstrated in melanoma patients, it offers biological plausibility for the taxon-level associations observed in the melanoma cohorts above. In contrast, dysbiosis-associated communities, including fungal overgrowth by *Candida* species, may impair epithelial barrier integrity, suppress immune activation, and promote T-cell dysfunction, reducing therapeutic efficacy [4,16,23].

Biomarker Consistency and Longitudinal Dynamics

Administration of a defined *F. prausnitzii* strain to antibiotic-perturbed mice restored sensitivity to anti-PD-1 blockade, supporting a causal role for microbial composition in treatment outcome [20]. Longitudinal human data suggest that microbiome dynamics may serve as early predictive biomarkers: loss of *A. muciniphila* during therapy has been associated with emerging resistance prior to clinical progression [2,15].

Confounders, Heterogeneity, and Clinical Translation

Substantial heterogeneity in study design, patient populations, and microbiome analysis methods limits generalizability [1,31]. Diet, antibiotic exposure, geographic variation, and host genetics are recurring confounders. Many studies are limited by small sample sizes and single-center designs, reducing statistical power and causal inference [21], and variability in sequencing platforms and analytical pipelines further complicates cross-study comparability [17,32]. Prior reviews have emphasized the need for standardized methodologies in microbiome research [33]. Integrative multi-omic approaches, combining metagenomic and metabolomic profiling, have identified specific microbial signatures and metabolites associated with response, reinforcing the value of functional characterization alongside taxonomic profiling [18,22,24].

Limitations

This review has several limitations. PubMed was selected as the primary biomedical database for this focused question, given its comprehensive indexing of the clinical and immuno-oncology literature relevant to melanoma, the gut microbiome, and immune checkpoint inhibitors. As additional databases such as Embase and Scopus were not searched and only English-language publications were included, some potentially eligible studies may not have been captured. Study selection, data extraction, and risk-of-bias assessment were conducted by a single reviewer. The included studies were predominantly small, single-center, observational cohorts with substantial heterogeneity in sequencing methodology and outcome definitions, limiting direct comparability. Risk-of-bias ratings of serious were assigned to eight of 25 studies, most often reflecting small sample size. Because of this methodological heterogeneity, a quantitative meta-analysis was not performed. Publication bias cannot be excluded because unpublished studies with negative findings may not have been identified.

Several statistical limitations further constrain interpretation. First, microbiome abundance data are compositional: taxon abundances are proportions of a constrained total, so an apparent increase in one taxon mechanically forces apparent decreases in others. Studies that applied standard tests to raw relative abundances, rather than compositionally aware methods (e.g., centered log-ratio transformation, ANCOM-BC (Analysis of Compositions of Microbiomes with Bias Correction), or ALDEx2 (ANOVA-Like Differential Expression 2)), are susceptible to inflated false-positive rates, and this susceptibility is inherited by any synthesis of their results. Second, differential-abundance analyses simultaneously test hundreds to thousands of taxa, making multiple-comparison correction decisive; because included studies varied in whether and how they controlled the false discovery rate, the taxa reported as associated with response are not equally robust across the evidence base. Third, the included studies are not statistically commensurable: they differ in sequencing approach (16S rRNA amplicon sequencing versus shotgun metagenomics), bioinformatic pipeline, taxonomic reference database, diversity indices, and differential-abundance method, for example LEfSe (Linear Discriminant Analysis Effect Size), DESeq2 (Differential Expression Analysis for Sequence Count Data 2), ANCOM (Analysis of Composition of Microbiomes), and Wilcoxon rank-sum, so effect estimates cannot be pooled without introducing an unquantifiable artifact. Clinical heterogeneity in the definition of response further compounds this. For these reasons, associations are reported by direction and consistency of effect rather than pooled magnitudes, and individual taxon-level findings should be regarded as hypothesis-generating.

A meta-analysis of effect estimates was not undertaken, and narrative synthesis was selected in accordance with the Synthesis Without Meta-analysis (SWiM) reporting guideline [34] and chapter 12 of the Cochrane Handbook for Systematic Reviews of Interventions [35], both of which specify narrative and other non-pooled synthesis methods as the appropriate approach when clinical, methodological, and statistical heterogeneity preclude the meaningful pooling of effect estimates. All three forms of heterogeneity are present here: clinically, studies differed in immune checkpoint inhibitor regimen and in the definition of treatment response; methodologically, they differed in sequencing strategy, bioinformatic pipeline, and differential-abundance testing; and statistically, they reported non-commensurable effect measures (e.g., linear discriminant analysis (LDA) scores, fold changes, and unadjusted or variably adjusted p-values) that cannot be combined on a common scale. Critically, the absence of a reproducible taxonomic signature across cohorts is itself a principal finding of this field rather than a limitation to be averaged away: even a unified reanalysis of pooled raw metagenomic data across cohorts found that microbiome-response associations were cohort-dependent and that no single species served as a consistent biomarker [2]. Pooling published summary statistics across incompatible pipelines would therefore generate a spuriously precise estimate that misrepresents the underlying evidence. Narrative synthesis, structured by direction and consistency of effect, was accordingly the methodologically sound choice.

## Conclusions

The gut microbiome is emerging as a modulator of immune checkpoint inhibitor outcomes in melanoma, influencing both therapeutic efficacy and immune-related toxicity through interactions involving microbial composition, metabolic function, and host immune response. Current evidence supports the potential of the microbiome as a predictive biomarker and therapeutic target, but findings remain heterogeneous and context-dependent, and most of the underlying evidence carries at least a moderate risk of bias. Large, prospective, multi-center, multi-omic studies that account for key confounding factors are needed before microbiome profiling can be integrated into clinical decision-making for melanoma immunotherapy.
